# Prospectively reinstated memory drives conscious access of matching visual input

**DOI:** 10.1038/s41598-019-41350-7

**Published:** 2019-03-18

**Authors:** Surya Gayet, Dirk van Moorselaar, Christian N. L. Olivers, Chris L. E. Paffen, Stefan Van der Stigchel

**Affiliations:** 1 Donders Institute for Brain, Cognition and Behaviour, Radboud University, Nijmegen, The Netherlands; 2 Amsterdam Brain and Cognition, Department of Psychology, University of Amsterdam, Amsterdam, The Netherlands; 3 Department of Experimental and Applied Psychology, Institute for Brain and Behavior Amsterdam, Vrije Universiteit Amsterdam, Amsterdam, The Netherlands; 4 Experimental Psychology, Helmholtz Institute, Utrecht University, Utrecht, The Netherlands

## Abstract

Maintaining information in visual working memory (VWM) biases attentional selection of concurrent visual input, by favoring VWM-matching over VWM-mismatching visual input. Recently, it was shown that this bias disappears when the same item is memorized on consecutive occasions (as memoranda presumably transit from VWM to long-term memory), but reemerges when observers anticipate to memorize a novel item on a subsequent trial. Here, we aimed to conceptually replicate and extend this intriguing finding, by investigating whether prospectively reinstated memory drives conscious access of memory-matching visual input. We measured the time it took for participants to detect interocularly suppressed target stimuli, which were either from the same color category as a concurrently memorized color or not. Our results showed that the advantage of memory-matching targets in overcoming suppression progresses non-monotonically across consecutive memorizations of the same color (‘repetitions’): the advantage for memory-matching visual input initially declined to asymptote, before being fully revived on the last repetition. This revival was not observed in a control experiment in which targets were not interocularly suppressed. The results suggest that, as observers anticipate to memorize a novel item imminently, VWM usage is prospectively reinstated, causing memory-matching visual input to gain accelerated access to consciousness again.

## Introduction

Visual working memory (VWM) is used to keep behaviorally relevant visual information available after termination of its retinal input. Oftentimes, the visual system has to process incoming visual input while at the same time maintaining previous visual input in VWM. These mnemonic and perceptual processes do not operate independently but rather cause preferential processing of visual input that matches the content of VWM^1–4^. Accordingly, it has been shown that, compared to memory-mismatching visual input, memory-matching visual input automatically attracts more attention^5–7^, automatically attracts more eye movements^7–11^, and is favored in bistable perception^12,13^. In addition, VWM appears to impact the processing of concurrent visual input at very early levels of visual processing, as perceptually suppressed visual input was shown to gain accelerated access to consciousness when it matches rather than mismatches the concurrent content of VWM^14–17^.

It goes without saying that VWM is not the only memory system at our disposal. With active learning through repeated memorization, memoranda presumably transit from VWM to long-term memory (LTM), as the neural mechanisms change from being activity-based^18^ to being plasticity-based^19–22^. Indeed, studies using electro-encephalography revealed that repeating the same memory item on a couple of successive trials suffices for the neural signature of VWM maintenance - the contralateral delay activity - to drop to asymptote, while the P170 amplitude – a signature of LTM usage – climbs to peak level^23–25^. Furthermore, Van Moorselaar, Theeuwes, and Olivers^26^ found that across repeated memorizations of the same item, attentional capture by memory-matching distractors decreases. This is in line with the idea that as memoranda are preserved in a less activity-based state, they influence concurrent visual processing to a lesser extent. Surprisingly, however, Van Moorselaar and colleagues^26^ also observed that memory-matching visual input started to capture attention again towards the end of a repetition sequence, just before observers had to memorize a new item on the next trial. When memory sequences were separated by an unrelated task (which did not involve memorizing a novel item), however, this memory-driven capture effect did not reemerge at the end of a sequence. The authors argued that, at the end of a sequence, observers anticipated to update the current memorandum with a new memory item, thereby causing the incidental reactivation of its representation in VWM. In line with this view, the data of Carlisle and colleagues^25^ (Figure 4D) also showed a numerical increase in the contralateral delay activity toward the end of a repetition sequence (although not formally tested).

In the present study, we sought to investigate whether prospectively reinstated VWM could favor conscious access of memory-matching visual input. Observing this would extend the finding of van Moorselaar and colleagues^26^ in two important ways. First, this would provide a conceptual replication (i.e., using a different probing method) of the surprising finding that VWM usage is reinstated when observers anticipate to memorize a novel item imminently. Second, this would demonstrate that prospectively reinstated VWM affects visual processing pre-consciously, as it co-determines the time-point of conscious detection. Thus, we hypothesized that the advantage of memory-matching (relative to memory-mismatching) visual input in gaining conscious access would progress non-monotonically over consecutive repetitions: this memory-contingent bias is expected to initially decline, as memory representations become less active with learning, and to be reinstated toward the end of a repetition streak, as observers anticipate to memorize a novel item imminently. Alternatively, this memory-driven bias could decrease monotonically, showing that prospectively reinstated memory does not affect conscious access.

**Figure 1:**
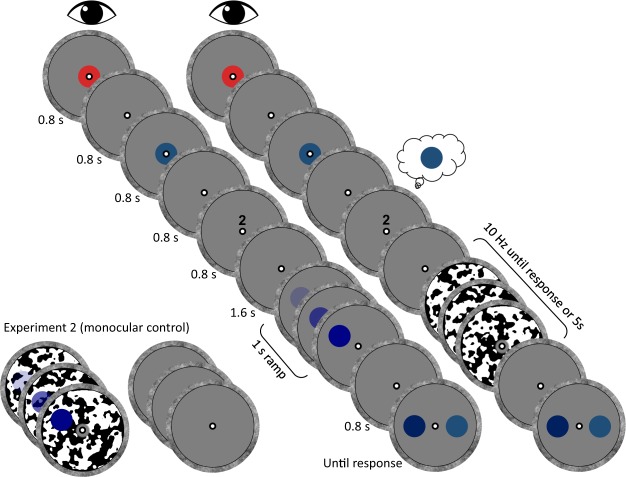
Schematic depiction of a trial in which the target matches the color category of the to-be-memorized item. On each trial, participants were sequentially presented with two consecutive memory items – drawn from a different color category – and a retro cue (“1” or “2”), indicating which item they should memorize for a subsequent recognition task. During the retention interval, participants were required to report the location (left or right of fixation) of a target that was either interocularly suppressed (Experiment 1) or not (Experiment 2). The target could either match the cued (i.e., memorized) memory item, or the non-cued (i.e., discarded) memory item. Critically, throughout the entire experiment, participants were required to memorize the exact same memory item during six consecutive trials (i.e., repetitions).

Figure 1 illustrates the procedure. On each trial, a retro cue instructed participants to memorize one out of two presented colors, such that one color was memorized and the other was discarded. During the retention interval, participants performed a breaking continuous flash suppression (b-CFS) task, in which they were required to report the location of an initially suppressed target as soon as they could discern its location (left or right of fixation). The b-CFS technique makes use of the fact that a target presented to one eye is initially suppressed by a dynamic masking pattern presented to the other eye (i.e., continuous flash suppression, or CFS)^27^. Eventually, the target overcomes the interocular suppression, and reaches conscious access. The time it takes for observers to report the initially suppressed target reflects the propensity of that target to gain conscious access and is therefore a proxy of the strength of the pre-conscious representation^28–30^. The color of the target was either drawn from the same color category as the memorized color (i.e., a memory-matching target), or was drawn from the same color category as the discarded color (i.e., a memory-mismatching target). Critically, participants were cued to memorize the same memory item for six consecutive trials. Existing evidence indicates that it suffices to memorize the same item on two or three successive trials to considerably reduce demands on the working memory system^24–26^. Response times to memory-matching and memory-mismatching targets were compared for each of the six repetitions, allowing us to investigate the progression of the memory-driven bias in conscious access during the waxing and waning of VWM usage.

This experimental setup provides two crucial assets for addressing our research question. Firstly, using b-CFS allows for isolating modulations of memory contents on visual processes that precede conscious access, from modulations of memory contents on visual processes that arise after conscious access. This is done by assessing the memory-driven response time benefit both for targets that are interocularly suppressed (as we do in Experiment 1), and for targets that are not (as we do in Experiment 2). This is important, as it allows for dissociating between perceptually driven influences of VWM maintenance on concurrent visual processing (i.e., caused by interaction with the mnemonic representation itself), from non-perceptual processes that are initiated after stimulus detection (i.e., driven by control processes; see General Discussion). Secondly, the current approach, in which the same item is memorized on consecutive trials, allows for varying VWM usage, while keeping both the task-instructions and the physical stimulation identical. Under these circumstances, a non-monotonic progression of the memory-driven bias in conscious access (i.e., initially waning before being reinstated in the last repetition) would provide evidence that prospectively reinstated memory drives conscious access of matching visual input.

## Methods

### Participants

Based on an optional Bayesian stopping rule (see the paragraph ‘stopping rule’), data of 36 participants were collected for Experiment 1 (12 males, with an average age of 21 years; *SD* = 1.9). This number of participants was then matched for Experiment 2 by collecting data from 36 new participants (13 males, with an average age of 22 years; *SD* = 1.7). The participant group consisted of undergraduate students recruited at Utrecht University. Participants provided informed consent before participating, and were compensated with either course credits or monetary reward upon completion of the experiment. The study was approved by the Ethics Committee of the Faculty of Social Sciences of Utrecht University, and all methods were performed in accordance with the ethical committee’s guidelines and regulations. All participants had (corrected-to) normal vision, and were tested for color blindness with the Ishihara color blindness test plates^31^, and tested for stereoscopic vision with the TNO test for stereoscopic vision^32^.

### Stimuli and apparatus

Participants were seated in front of a mirror stereoscope mounted on a chinrest, which ensured separate stimulation of the two eyes, at an effective viewing distance of 57 cm to a linearized CRT monitor (22″ LaCie Electron Blue IV, 1024 by 768 pixels). Responses were collected using the arrow keys of an Apple keyboard, and the temporal resolution of response time registration was limited by the refresh rate of the monitor (100 Hz). In order to promote binocular fusion of the complementary images, identical Brownian noise frames were presented to both eyes, delimiting the gray presentation area on which all stimuli were presented. The presentation area consisted of a circular region with a diameter of 4.5 degrees of visual angle (dva). At all times, a black (<0.1 Cd/m^2^; 0.2 dva) and white (32 Cd/m^2^; 0.1 dva) fixation bullseye was presented at the center of the presentation areas.

The memory items used for the memory task, and the target stimuli used for the b-CFS task, consisted of colored disks with a diameter of 0.6 dva. The memory items were centrally presented, and had a mean luminance of 3.35 Cd/m^2^. They were retrieved from one of our earlier studies^16^, but were extended from four to five colors per category (see Table 1), so that each color would be used fewer times throughout the experiment. In addition, a separate b-CFS pilot study (N = 8) was used to adaptively adjust the luminance output of the red, green, and purple target stimuli, until they elicited comparable detection times as the blue reference stimulus on our set-up. Finally, the luminance of the gray background was chosen such as to match the average luminance of the target stimuli, so that the target stimuli were primarily defined by their chromatic contrast rather than their luminance contrast with the background. Target stimuli were increased from 0 to 100% opacity (i.e., linear addition of target stimulus and background contribution) in one second, and were presented at a fixed eccentricity of 2.25 dva, left or right of fixation, randomly jittered within 30 degrees of the horizontal midline. The retro-cues – indicating which of two successively presented memory items should be memorized for a subsequent recognition task – consisted of the Arabic numerals “1” or “2” written in a black Arial font (0.6 dva).

**Table 1:** Overview of the stimulus colors (CIE values*) used in Experiments 1 and 2.

Color	X-value	Y-value	Luminance (Cd/m^2^)
Red 1	0.595	0.363	3.84
Red 2	0.619	0.349	3.54
Red 3	0.651	0.338	3.75
Red 4	0.570	0.315	3.47
Red 5	0.582	0.326	3.15
Average (SD)	0.603 (0.032)	0.338 (0.018)	3.55 (0.27)
Green 1	0.270	0.529	3.22
Green 2	0.281	0.596	3.43
Green 3	0.292	0.608	3.69
Green 4	0.307	0.584	3.14
Green 5	0.328	0.555	2.99
Average (SD)	0.296 (0.023)	0.574 (0.032)	3.29 (0.27)
Blue 1	0.165	0.135	3.44
Blue 2	0.159	0.106	3.35
Blue 3	0.147	0.121	3.50
Blue 4	0.152	0.080	2.86
Blue 5	0.171	0.092	3.15
Average (SD)	0.159 (0.010)	0.107 (0.022)	3.26 (0.26)
Purple 1	0.226	0.123	3.41
Purple 2	0.249	0.133	3.28
Purple 3	0.271	0.141	3.28
Purple 4	0.292	0.152	3.39
Purple 5	0.284	0.184	3.15
Average (SD)	0.264 (0.027)	0.145 (0.023)	3.30 (0.10)
Red target	0.608	0.341	3.83
Green target	0.293	0.579	4.15
Blue target	0.157	0.102	2.86
Purple target	0.263	0.150	3.71
Gray background	0.311	0.329	3.63

*CIE values stands for Commission Internationale d’Eclairage values, as measured from viewing distance (i.e., 57 cm) with a PR-650 SpectraScan colorimeter/telephotometer (Photo Research, Inc.).

The masks used for eliciting CFS were created by filtering pink (1/f) noise using a rotationally symmetric Gaussian low-pass filter (σ = 3.5) and making the resulting image binary (black and white, >99% Michelson contrast). The same masks were used in earlier studies from our labs^14,16,17^. On every trial, 20 new masks were generated, which were presented for 100 ms each (10 Hz) in random order, with the restriction that the same mask was never presented twice in succession.

### Experimental procedure

Before the experiment was initiated, participants performed a standard b-CFS task (with saturated blue disks as target stimuli) in order to determine eye-dominance within the b-CFS paradigm. Throughout the rest of the Experiment, the target was presented to the non-dominant eye (in both Experiment 1 and Experiment 2). The CFS masks were presented to the other eye in Experiment 1 (thereby eliciting interocular suppression), and to the same eye as the target in Experiment 2 (thereby eliciting no interocular suppression). Next, participants performed 18 practice trials to get acquainted with the experiment. Finally, the experiment was initiated, constituting of 192 trials divided into 12 blocks of 3 minutes each.

The sequence of events in a trial is depicted in Fig. 1. Each trial started with a blank (i.e., the gray presentation area with a black-white fixation bulls-eye, for 1000 ms), a central disk with a color drawn from one of the four color categories (800 ms), a blank (800 ms), a central disk with a color from the complementary color category (800 ms), a blank (800 ms), and the number “1” or “2” (800 ms), which instructed participants to memorize either the first or the second stimulus. After another blank (1600 ms), the CFS masks were presented at 10 Hz and, after a variable delay ranging between 300 and 600 ms, the target was gradually increased from zero to full opacity over the course of one second. The presentation of target and dynamic masks was continued for a maximum of five seconds, or until participants provided a response. Participants were instructed to report as fast and accurately as possible on which hemifield (i.e., left or right of fixation) the target appeared. Next, three more masks were presented dichoptically (300 ms) to eliminate after images, followed by a blank (800 ms), after which two colored discs were presented for the recognition task: one disc was of the exact same color as the cued memory item, and the other disc was of a different color (but of the same color category). Participants were required to provide a non-speeded report as to which of these two colors was identical to the one they were cued to memorize before.

### Experimental design

The experimental design for both experiments comprised two within-subject factors of interest: the factor Repetition with six levels, (repetition one to six) and the factor Congruence with two levels (the target matches the color of the memorized or of the discarded memory item). All combinations of these factor levels were equally prevalent within each experimental block. Within-subject factors of non-interest that were fully counterbalanced with the factors of interest included the color category of the cued memory item (red, blue, green, or purple), the retro-cue (“1” or “2”), and the hemifield on which the target was presented (left or right of fixation). Within-subject factors of non-interest that were not counterbalanced (but for which equal prevalence was maximally approximated) included the exact hue of the cued memory item (one of five), the exact hue of the discarded memory item (one of five), the exact location of the target stimulus (i.e., one of 12 angular positions per hemifield), the location of the correct response during the recognition task (left or right), and the exact hue of the distractor during the recognition task (one of the remaining four hues within the color category of the cued memory item).

A critical aspect of the current experimental paradigm is that the experiment was subdivided into sequences of six consecutive trials (i.e., repetitions) in which the same memory item was cued for memorization. The item that was not cued for memorization was also of the exact same hue across the six repetitions, but all other factors were varied (i.e., the target location, the retro-cue, target congruence, and the hue and location of the distractor during the memory task). Repeating the discarded memory item across repetitions as well, allowed for isolating the influence of repeated memorization (which only occurs for the to-be-memorized item) from repeated stimulation (which occurs for both the discarded and the memorized memory items) on the processing of concurrent visual input.

Color categories were paired, so that when a red memory item was cued, the non-cued (i.e., discarded) item was always of the blue color category (and vice versa), and when a green memory item was cued, the non-cued item was always of the purple color category (and vice versa). This was done to reduce the number of conditions, and to avoid spurious categorizations between purple, red, and blue color variations. All trials were presented in a pseudo-randomized order, with the only restriction that participants were never cued to memorize an item from the same color category in successive series of repetitions. This was done to ensure that memory items on the first repetition were always clearly dissociable from the previously memorized item (i.e., the last repetition of the previous series of six consecutive trials). Participants were not informed about the occurrence (or number) of memory repetitions in the experiment.

### Monocular control (Experiment 2)

Response times to interocularly suppressed targets are composed of (1) the duration of interocular suppression, and (2) the response speed after the interocular suppression is resolved. Experiment 2 was aimed at assessing whether (in Experiment 1) the memory content genuinely affected interocular suppression durations, or whether the memory content elicited differences in response speed arising after the interocular suppression was resolved (or both). For this purpose, the target and masks were now presented to the same eye, so that no interocular suppression was induced. The rationale behind this so-called monocular control experiment is that any influence of (in this case) memory content on interocular suppression durations should be abolished (as there is no interocular suppression), but any influence of memory content on processes that arose after the interocular conflict was resolved, should be preserved (this approach has been extensively discussed in the literature^29,30,33^).

It should be noted that the current implementation of the monocular control condition does not fully follow the guidelines set out by Gayet *et al*.^30^, in which (1) we argued in favor of intermixing interocular suppression trials and monocular control trials, and (2) we advocated the usage of two monocular control conditions: one that matches the stimulus timing, and one that matches the response times of the interocular suppression condition. We refrained from choosing this optimal set-up in light of two considerations. Firstly, this would lead to a three-fold increase in the number of trials per participant, and thereby to overlearning of the different hues that were used for the memory task. This is potentially problematic, as overlearning of memory items might reduce participants’ need to rely on VWM storage for performing the delayed match-to-sample task. Secondly, we deemed it unnecessary to employ this optimal set-up, because our main goal was not to demonstrate whether or not memory maintenance affects processing of visual input prior to conscious access per se (this has already been established in earlier work^13,17,34^), but to investigate whether the influence of memory maintenance on the processing of concurrent visual input varies with learning, as observers are repeatedly exposed to the same memorandum.

### Stopping rule

In order to ascertain sufficient experimental sensitivity, we used an optional Bayesian stopping rule: we decided a priori that we would test until a Bayes Factor of 6 was observed for both the first and last repetitions, in favor of either the null hypothesis (i.e., no influence of VWM on response times, BF_+0_  < $\frac{1}{6}$), or the alternative hypothesis (i.e., faster response times to memory-matching targets, BF_+0_ > 6), with a minimum of 20 participants. Bayes Factors were computed in JASP^35^, using the standard Cauchy prior width of 0.707. Because, to the best of our knowledge, all behavioral effects of VWM contents on concurrent visual input reported in the literature reflect a bias towards memory-matching visual input, we opted for directional Bayesian tests. In order to maintain comparable experimental power between experiments, we based the number of participants in Experiment 2 on the number of participants obtained through the stopping rule in Experiment 1, thereby allowing us to conduct additional explorative between-group analyses.

### Data analyses

The critical comparisons consisted of the differences in response times between memory-matching and memory-mismatching targets, for each of the six repetitions. Any difference that is observed between these conditions can only be explained by memory-based modulations of the concurrent visual input. This follows from the fact that, across all trials, matching and mismatching trials elicit the exact same visual input, and the exact same memory content, but only differ in terms of the contingency between the memory content and visual input. Part of the variance in response time difference between these conditions can be explained by individual differences in absolute response times^33^, which is of no interest for the current research question. To remove this variance of non-interest, we conducted statistics on latency-normalized response time differences between memory-matching and memory-mismatching conditions. This metric is obtained by dividing the median response time difference between the memory-matching and memory-mismatching conditions, by the averaged median response times in the memory-matching and memory-mismatching conditions. Thus, the latency-normalized response time difference describes by what fraction response times to a target are reduced when it matched rather than mismatched the concurrent memory content. This normalization procedure has the additional advantage of yielding a pattern of response time differences that more closely approximates a normal distribution^33^, so that parametric tests can be applied. Shapiro-Wilkinson tests of normality confirmed that the assumption of normality was not violated in any of the repetition conditions after latency-normalization (all *p*’s > 0.05). For all tests, a Bayes factor above 3 for either the null or the alternative hypothesis was regarded as substantial evidence in favor of that hypothesis^36–38^.

The Bayesian *t*-tests on the RT difference between memory-matching and memory-mismatching targets were not corrected for multiple comparisons across the different repetitions of the same memorandum. This follows three main considerations. First, Bayes factors are based on posterior odds, which reflect the belief in one’s hypotheses *after* having observed the data (i.e., the extent to which the data more closely resembles what the data would have looked like under hypothesis A or under hypothesis B). The interpretation of Bayes factors is therefore unaffected by the number of statistical tests that are conducted^39–41^. Second, the data in consecutive repetitions are not independent; if anything, consecutive repetitions with a relatively feeble effect in the same direction, provide stronger (rather than weaker) evidence for that effect. Third, we formulated clear predictions regarding the progression of the memory-based bias in conscious access across repetitions (i.e., non-monotonic, monotonic, or flat progression). Aside from the Bayesian *t*-tests on each repetition, these predictions were also explicitly pitted against one another by comparing regression models that best fit the pattern of observed data.

## Results

### Experiment 1 (interocular suppression)

First, we aimed to assess whether we could replicate Gayet *et al*.’s^13^ findings that the location of initially interocularly suppressed targets can be reported faster when they match compared to when they mismatch the concurrent memory content, irrespective of (i.e., collapsed over) the repetition of memory items. A directional Bayesian paired-samples *t*-test revealed that it was 32 times more likely that the observed data reflected faster response times for memory-matching relative to memory-mismatching targets, than reflecting equal response times for both, BF_+0_ = 32.3. Overall, observers were 4.2% faster on memory-matching than on memory-mismatching trials (*SD* = 7.7%; corresponding to 61 ms).

**Figure 2:**
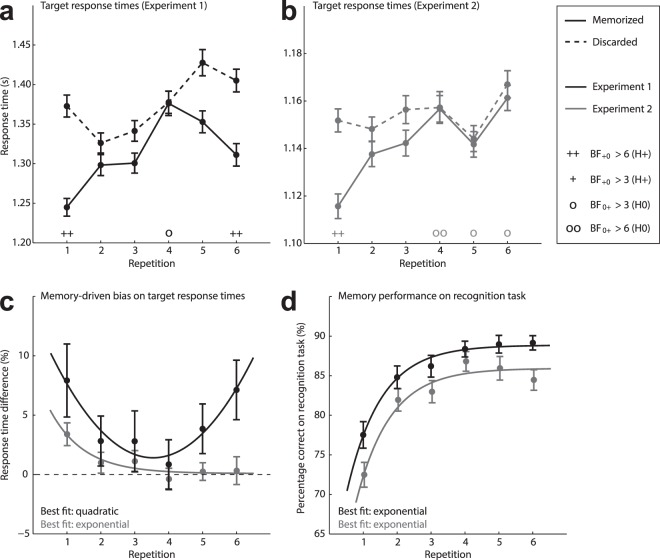
Results of Experiment 1 (black) and Experiment 2 (gray). Panels (a,b) depict the raw response times to the target presented during the retention interval, depending on whether it matched (i.e., memorized condition; solid line) or mismatched (i.e., discarded condition; dashed line) the color category of the memorized item. Bayesian support for the null (o) or alternative hypothesis (+) is provided for each repetition is provided directly above the corresponding label on the x-axis. Panel (c) depicts the percentage decrease in response time to memory-matching (memorized condition) relative to memory-mismatching (discarded condition) targets. Panel (d) depicts participants’ recognition accuracy on the recognition task following the retention interval. The goodness of fit of different regression models was compared using Akaike Information Criterion (AIC) values. Error bars represent standard errors of the mean.

Next, we separately investigated the decrease in response times for memory-matching targets on each successive repetition of the same memory item, using directional Bayesian *t*-tests (see Fig. 2, Panel a). On the first repetition, memory-matching targets were responded to 7.9% (*SD* = 18.5%) faster than memory-mismatching targets (corresponding to 128 ms), BF_+0_ = 6.2. The second and third repetitions yielded insufficient evidence to either support or refute a difference in response times between memory-matching and memory-mismatching targets, $\frac{1}{3}$ < BF_+0_ < 3 (28 ms and 41 ms faster response to memory-matching targets respectively). On the fourth repetition, substantial evidence was observed in favor of the null hypothesis, BF_0+_  = 4.0, showing that the difference in response times between memory-matching and memory-mismatching trials had vanished (1 ms faster response to memory-matching targets). The fifth repetition again yielded insufficient evidence to either support or refute a difference in response times between memory-matching and memory-mismatching targets, $\frac{1}{3}$ < BF_+0_ < 3 (75 ms faster response to memory-matching targets). Crucially, the influence of memory content on response times was revived on the sixth repetition, as memory-matching targets were again responded to 7.1% (*SD* = 15.0%) faster than memory-mismatching targets (corresponding to 94 ms), BF_+0_ = 10.8. As such, the memory-driven bias in conscious access of matching visual input initially diminished across early repetitions, but was reinstated on the last repetition as observers could anticipate to memorize a novel item on the subsequent trial.

In order to test whether the progression of response time differences across successive repetitions is indeed best explained by a U-curve, we fitted monotonic (linear, exponential) and non-monotonic (quadratic, cubic) regression models to the latency-normalized response time differences across repetitions. Akaike Information Criterion (AIC) values were used to compare the goodness of fit (with lower numbers representing better fit), while penalizing for the number of included parameters^42,43^. The left column of Table 2 shows that the quadratic regression (closely followed by the cubic regression) best describes the data, clearly outperforming the two monotonic regression models. We next assessed the relative likelihood of one model over another (e.g., RL_M1>M2_) to minimize information loss, following the equation: ${{\rm{RL}}}_{{\rm{M}}1 > M2}={{\rm{e}}}^{0.5\ast ({{\rm{AIC}}}_{M2}-{{\rm{AIC}}}_{M1})}$. This showed that the quadratic regression model was 299 times more probable to minimize information loss than the exponential regression model, and 812 times more probable to minimize information loss than the linear regression model (see Fig. 2, Panel c). This comparison of regression models corroborates the findings obtained with Bayesian *t*-tests, described earlier, and further establishes that the memory-driven bias in conscious access initially declined before reemerging toward the end of a repetition streak.

**Table 2:** Akaike criterion values for the goodness of fit of (non-)monotonic regression models.

Fit		Experiment 1		Experiment 2	
		Target detection	VWM performance	Target detection	VWM performance
Monotonic	Linear	34.0	30.8	19.7	36.1
	Exponential	32.0	14.8*	11.6*	23.5*
Non-monotonic	Quadratic	20.6*	22.6	14.2	25.9
	Cubic	22.4	19.5	15.6	26.4

Note. Asterisks denote the regression model that best fits the observed data.

### Experiment 2 (monocular control)

Experiment 2 was aimed to assess whether the prospective reinstatement of the memory-driven bias in Experiment 1 actually reflected processes that occur before or after the interocular conflict is resolved. Again, we first investigated whether response times to memory-matching and memory-mismatching targets differed, with response times collapsed across repetitions. Subsequently, response time to memory-matching and memory-mismatching targets were compared for each repetition separately.

In contrast to earlier findings (e.g., Experiments 2 and 3 of Gayet *et al*.^13^; but see Experiment 1B of Van Moorselaar *et al*.^17^) we observed a small but robust effect of memory content on response times in the monocular control experiment. A directional Bayesian paired-samples *t*-test revealed that it was 6 times more likely that the observed data reflected faster response times for memory-matching compared to memory-mismatching targets, than reflecting equal response times for both, BF_+0_ = 5.8. Overall, observers were 1.0% faster on memory-matching than on memory-mismatching trials (*SD* = 2.2%; corresponding to 11 ms). A Bayesian independent-samples *t*-test between Experiment 1 and Experiment 2 provided substantial evidence that the impact of memory content on response times is more pronounced in the case of interocular suppression than in the absence of interocular suppression (61 ms or 7.7%, compared to 11 ms or 1.0%), BF_+0_ = 6.2. This suggests that, across all repetitions, memory contents affect processes that emerge after the interocular conflict is resolved (Experiment 2), as well as interocular suppression durations (Experiment 1).

In order to investigate how the influence of memory content on detection times evolved over successive repetitions, we conducted directional Bayesian paired-samples *t*-tests for each of the six repetitions (see Fig. 2, panel b). This revealed that, on the first repetition, memory-matching targets were responded to 3.4% (*SD* = 5.8%) faster than memory-mismatching targets (corresponding to 36 ms), BF_+0_ = 51.8. The second and third repetitions yielded insufficient evidence to either support or refute a difference in response times between memory-matching and memory-mismatching targets, $\frac{1}{3}$ < BF_+0_ < 3 (11 ms and 14 ms faster response time to memory-matching targets respectively). On the fourth, fifth, and sixth repetition, the data provided substantial evidence for the null hypothesis that response times to memory-matching and memory-mismatching targets did not differ; BF_0+_  = 7.6, BF_0+_  = 4.3, and BF_0+_  = 4.5 respectively (1 ms, 2 ms, and 6 ms faster response time to memory-matching targets respectively). As such, memory-matching targets were reported faster than memory-mismatching targets on the first repetition, even in the absence of interocular suppression. In contrast to Experiment 1 in which targets were interocularly suppressed, however, this effect was not revived on the last repetition in the absence of interocular suppression.

To further investigate this qualitative difference between experiments, we conducted Bayesian independent-samples *t*-test to assess whether the memory content affected response times differently in the presence (Experiment 1) or absence (Experiment 2) of interocular suppression. These tests revealed that the evidence for a difference between Experiments 1 and 2 was inconclusive for repetitions one to five (i.e., $\frac{1}{3}$ < BF_+0_ < 3), but on the last repetition the memory content impacted response times to a greater extent in the presence (7.1%, *SD* = 15.0%; corresponding to 94 ms) than in the absence (0.3%, *SD* = 7.0; corresponding to 6 ms) of interocular suppression, BF_+0_ = 6.1. A comparison of regression models on the data of Experiment 2 (see Table 2) confirmed that the observed difference in response times between memory-matching and memory-mismatching conditions in the absence of interocular suppression is best described as a monotonic (exponential) decrease over successive repetitions. Computing the relative likelihoods of pairs of models revealed that the exponential regression model was 4 times more probable to minimize information loss than the quadratic regression model, and 7 times more probable to minimize information loss than the cubic regression model (see Fig. 2, Panel c). Taking together the findings of Experiments 1 and 2, these data demonstrate that prospectively reinstated VWM maintenance effectively reduced the suppression duration (and thus enhanced conscious access) of matching visual input.

### Recognition task (Experiments 1 and 2)

The main purpose of analyzing participants’ performance on the recognition task was to verify whether they performed above chance level, thereby establishing that they complied with the task demands (i.e., memorizing the cued memory item). This was analyzed using a directional Bayesian one-sample *t*-test against the 50% chance level. Collapsed over all six repetitions, participants were 85.8% (*SD* = 5.5) accurate in reporting which color they had been cued to memorize on that trial in Experiment 1, which is higher than the 50% chance level, BF_+0_ = 1.8*10^27^. Similarly, in Experiment 2, participants were 82.4% (*SD* = 6.7) accurate in reporting which color they had been cued to memorize on that trial, which is higher than the 50% chance level, BF_+0_ = 1.3*10^23^. A Bayesian independent-samples *t*-test revealed that the difference in memory recognition accuracy between Experiments 1 and 2 was statistically unreliable, $\frac{1}{3}$ < BF_+0_ < 3.

A secondary purpose of analyzing performance on the recognition task was to establish that task performance on the recognition task increased monotonically across repetitions. This is important, because, if performance on the recognition task follows the same non-monotonic pattern as the response time differences analyzed above, then task difficulty (on the recognition task) rather than VWM usage could have caused response times differences to vary across repetitions. This was not the case: For Experiments 1 and 2, an exponential regression fit best explained the increase in performance on the recognition task over successive repetitions of the same memory item. A comparison of AIC values for the different regression models is provided in Table 2, and the winning regression fit is depicted in Fig. 2 (Panel d). Specifically, the exponential regression models (for Experiments 1 and 2 respectively) were 49 and 3 times more probable to minimize information loss than the quadratic model, and 10 and 4 times more probable to minimize information loss than the cubic model. Considering that VWM performance increases monotonically across repetitions, it can account for the waning of the memory-driven bias across repetitions in Experiment 2, where targets were not interocularly suppressed. In contrast, VWM performance cannot account for the non-monotonic progression of VWM influence on concurrent visual input, as observed in Experiment 1, where targets were interocularly suppressed. Hence, the reemergence of the memory-driven bias in conscious access is caused by a change in VWM usage, rather than VWM precision.

### Target localization task (Experiments 1 and 2)

The performance on the left/right target location discrimination task was 98.2% (*SD* = 2.7) in Experiment 1, and 99.3% (*SD* = 1.0) in Experiment 2. Performance on the target localization task did not reliably differ between experiments, as evidenced by a non-directional independent-samples Bayesian *t*-test, $\frac{1}{3}$ < BF_+0_ < 3.

## Discussion

Previous studies showed that visual input gains accelerated conscious access when it matches the concurrent memory content^14–17^. Here, we instructed participants to memorize the same item in series of six consecutive trials (repetitions), to investigate how repeated memorization would affect this memory-driven bias in conscious access. To summarize our results: At the first presentation of a memorandum (repetition one), we found an advantage for reporting memory-matching over memory-mismatching targets in both the interocular suppression experiment and the monocular control experiment, and the statistical test was inconclusive as to whether or not there was a difference between experiments. As such, we cannot assert whether this effect was brought about before or after the interocular competition was resolved (or both). From repetitions two to five, there was no influence of the memoranda on response times (and thus on conscious access) at all, neither before nor after the interocular competition was resolved. At the end of a sequence (repetition six), the advantage for reporting memory-matching targets reemerged in the interocular suppression experiment, thus providing a conceptual replication of the rebound effect recently observed by Moorselaar and colleagues^26^. Surprisingly, this reemergence was not observed in the monocular control experiment. Substantial evidence for the null hypothesis in this experiment (no difference between memory-matching and memory-mismatching targets) thus demonstrates that the reemergence observed in the interocular suppression experiment is indeed specific for interocular suppression (all other things between Experiments 1 and 2 being equal). Consequently, we conclude that prospectively reinstated memory drives conscious access of memory-matching visual input. This pattern of findings suggests that VWM activity was reinstated when observers anticipated to memorize a novel item on the subsequent trial, causing the preferential conscious access of memory-matching visual input to reemerge.

The present findings are in line with those of van Moorselaar and colleagues^26^, who demonstrated that the influence of memory maintenance on attentional capture was revived when observers planned to memorize a novel item on the subsequent trial. Our current work adds to the findings of Van Moorselaar *et al*.^26^ in two main ways. Firstly, we extend these findings by showing that the reinstatement of this memory-driven bias was observed under conditions of interocular suppression (Experiment 1), but not in the absence of interocular suppression (Experiment 2). As such, prospectively reinstated memory impacts visual input that is not yet consciously accessible (this follows from the fact that memory affected the point in time at which the visual input became consciously accessible). This strongly suggests that the bias toward memory-matching visual input that reemerged toward the end of a repetition streak is perceptual in nature (e.g., driven by reinstatement of VWM activity), rather than reflecting a response bias (which should have affected response times in the monocular Experiment 2 as well). The present data thus corroborates the hypothesis formulated by Van Moorselaar *et al*.^26^: the anticipation of memorizing a novel item causes reinstatement of the VWM machinery, as a result of which the current memorandum is transferred back into an activity-based storage state, and interacts with the processing of concurrent visual input again (this is further elaborated below). Secondly, the current study provides a conceptual replication of the finding of Van Moorselaar *et al*.^26^. That is, we observe the prospective reinstatement of the memory-driven bias in a different paradigm, involving different visual stimulation, and a different response task. Also, in their study, the reinstatement occurred on either repetition 3 or repetition 9 (depending on which repetition was the last in that particular experiment), and in our study this occurred on repetition 6 (the last repetition in our design). In that regard, the current study adds to the evidence that the putative reinstatement of the VWM machinery is flexible and depends on the observer’s expectation of imminent VWM usage (i.e., to memorize a new color). While these latter points might seem trivial, we believe they are of utmost importance, considering that extraordinary claims require extraordinary evidence, and thus help to establish this intriguing finding.

The reinstatement of the memory-bias on the last repetition leaves to wonder why the anticipation of memorizing a novel item would cause the current memorandum to influence concurrent perception again. This is particularly striking, given that, after multiple repeated memorizations, memoranda no longer affect perception (as observed in our study) and no longer evoke contralateral delay activity^23–25^, and thus no longer seem to be represented in an activity-based VWM state. Surely, the anticipation of memorizing a novel item does not reinstate all items that observers have ever stored in LTM, as this would also include the non-cued (discarded) color, and a plethora of other unrelated (e.g., childhood) memories. Yet, the anticipation of memorizing a novel item did collaterally reinstate the current memorandum in a state that caused it to affect perception once again. This strongly suggests that, unlike other LTM content, information about the current memorandum was somehow preserved in VWM storage sites, but in a state that is not (as much) reflected in the delay activity, and did not affect perception. Such states have recently been shown to exist for so-called unattended memory items: these are memoranda that are preserved for later recall, but are sidelined by a cue indicating imminent recall of another memorandum^44^. An unattended memory item is not (or less) reflected in delay activity, but stimulus-specific delay activity representing the unattended memory item can be reinstated by stimulating the involved neural populations in a stimulus a-specific manner (i.e., ‘pinging the brain^45^). This suggests that some information about the unattended memory item was somehow retained in the VWM storage site, in an activity silent^45^ or dampened^46^ state. Considering the current study, it is conceivable that a memorandum requires gradually less attentional resources with repeated memorization (i.e., memory performance increases across repetitions), as a result of which delay activity decays, but a trace is preserved in VWM storage sites. In this scenario, the reactivation of VWM control mechanisms (for imminent memorization of a novel item) collaterally activates the VWM storage site in a stimulus-aspecific manner, thus coincidentally boosting the (stimulus-specific) representation of the memorandum, such that it once again biases concurrent perception. Future research should reveal whether, after repeated memorization of the same memorandum, some information about the memorandum is indeed preserved in VWM storage sites.

The discrepancy between Experiments 1 and 2 raises the question why prospectively reinstated memory would selectively affect visual input when it is interocularly suppressed. Could a difference in experimental sensitivity between Experiments 1 and 2 cause a spurious difference between the two experiments? This relates to a current debate on the overall effectiveness of the monocular control condition in isolating post-suppression effects^29,33^. Considering that well-established effects (such as faster detection of upright compared to inverted faces) were sometimes not observed in monocular control conditions^28,29^, one could wonder whether the monocular control condition is suitable for uncovering post-suppression effects. Experimental insensitivity is unlikely to explain the current results, however, as the faster response times to memory-matching visual input on repetition 1 was statistically more robust in Experiment 2 (without interocular suppression) than in Experiment 1 (with interocular suppression). Therefore, if anything, the monocular control Experiment appears to be more sensitive in uncovering differences in response times between memory-matching and memory-mismatching visual input than the interocular suppression experiment. Additionally, the usage of Bayesian analyses further confirmed that the null effect on repetition 6 in Experiment 2 reflected genuine evidence for the absence of a difference in response times, rather than reflecting absence of evidence (i.e., experimental insensitivity). With the issue of experimental sensitivity now brushed aside, it is crucial to evaluate what we are exactly measuring in Experiments 1 and 2. In the monocular control experiment, in which no interocular suppression was induced, the advantage for reporting memory-matching over memory-mismatching targets could be driven by non-perceptual effects (e.g., a more liberal response tendency for reporting memory matching targets) or perceptual effects (e.g., increased perceptual sensitivity to memory-matching targets). Bearing in mind that Experiments 1 and 2 only differed in the occurrence of interocular suppression, any effect that influenced response times in the monocular control experiment, should similarly influence response times in the interocular suppression experiment (but after the interocular conflict is resolved, hence ‘post-suppression effects’). Conversely, effects that are observed in the interocular suppression experiment but not in the monocular control experiment reflect a difference in interocular suppression duration (hence ‘pre-suppression effects’): shorter interocular suppression durations for memory-matching visual input. On the last repetition, we observed that the pre-suppression effects were revived (this follows from the presence of an effect in Experiment 1, given a null effect in Experiment 2), but surprisingly the post-suppression effects were not (this follows from the null effect in Experiment 2). We can conceive of at least two distinct ways in which VWM content can enhance responses to matching visual input. Firstly, sensory representations (from visual input) could be enhanced when they match concurrent mnemonic representations (maintained in VWM), as they draw upon a shared neural substrate^3,4,16,44–51^, thus allowing for faster perceptual reports to memory-matching visual input. If it is indeed the case that the current memorandum is reinstated in an activity-based VWM state on the last repetition, the memory-driven bias toward memory-matching visual input is expected to reemerge on the last repetition (as was observed in the interocular suppression experiment). Secondly, VWM maintenance could cause a decisional (i.e., post-suppression) bias toward memory-matching visual input, so that participants employ a more liberal response tendency for reporting upon memory-matching compared to memory-mismatching visual input. An interpretation of the current findings is that observers are more prone to such decisional biases toward memory-matching visual input, when memorization requires more effort, and thus more attentional resources were devoted to the memorandum. In line with this view, the memory-driven bias in the monocular control experiment decreased monotonically, while the performance on the memory recall task increased monotonically. In sum, the present paradigm potentially allows for reviving activity-based VWM storage, without reviving decisional biases that accompany effortful memory encoding. Future research is needed, however, to explicitly test whether the post-suppression component indeed co-varies with the amount of attentional resources allocated to VWM encoding, while the pre-suppression component depends on activity-based memory storage.

The present data also provided a conceptual replication of another important finding: the perceptual bias towards memory-matching visual input declines when the same item is repeatedly memorized. In our study, the response time benefit for memory-matching targets declined to asymptote over the course of the first handful of repetitions, in both the interocular suppression experiment (Experiment 1) and the monocular control experiment (Experiment 2). Thus, after a handful of repetitions, there was no more influence of memory content on the processing of concurrent visual input. Because, the six repetitions were identical in terms of visual stimulation and task instructions, the only difference is the number of times that a particular item had been successively memorized, presumably causing the memorandum to transit from VWM to LTM storage^21,22,25^. As such, this finding shows that VWM maintenance is required (and LTM usage is not sufficient) for accelerating conscious access of memory-matching visual input. An alternative view, however, is that it is not VWM maintenance per se (in the form of delay activity), but task-relevance that causes accelerated conscious access of memory-matching visual input. That is, when using a memory-recall task, memory-matching and memory-mismatching targets differ in two ways: (1) the memory-matching target matches a concurrently memorized feature, and (2) the memory-matching target matches a feature that is relevant for an upcoming task. This distinction is important because, visual input that is behaviorally relevant to the observer can have an advantage in overcoming interocular suppression, even in the absence of (explicit) memorization^30^. For instance, face stimuli with gaze directed toward the observer overcome interocular suppression faster than those with averted gaze^52^, and visual stimuli associated with reward overcome interocular suppression faster than stimuli that are neutral or that are associated with threat^53–56^. Similarly, a visual feature that is coincidentally relevant for a secondary task – performed in close temporal proximity – is more likely to overcome interocular suppression compared to a visual feature that is irrelevant for this secondary task^57^, even when no VWM maintenance is required. The present paradigms allows to dissociate between the roles of VWM retention and of behavioral relevance, because the relevance of the memorized color category for the upcoming memory task remains unchanged across repetitions, while VWM usage varies^23–25^. Thus, the present data confirms earlier claims that VWM maintenance drives the accelerated conscious access of memory-matching visual input^14–17^, by demonstrating that behavioral relevance of the memorized feature category alone cannot explain memory-driven biases in conscious access.

Perhaps surprisingly, the faster response times to memory-matching relative to mismatching targets on the first presentation of a memorandum (Repetition 1), was observed not only in the interocular suppression experiment (Experiment 1) but also in the monocular control experiment (Experiment 2). As such, we cannot conclude from the present data alone whether the response-time benefit for memory-matching targets on Repetition 1 was caused by (A) reduced suppression durations for memory-matching targets, or whether (B) this difference was initiated after the targets were released from suppression. In general, comparing the magnitude of response time differences between conditions with and conditions without interocular suppression is problematic, because of the inherent differences in visual stimulation and perceptual experience between the two conditions^29,33^. As such, the current study was not set-up to detect such a difference between experiments. Rather, we were interested in qualitative differences between said conditions across memory repetitions. The data revealed such a qualitative difference across memory repetitions: response time benefits for memory-matching targets decreased monotonically in the Experiment without interocular suppression, and progressed non-monotonically in the Experiment with interocular suppression, in which a ‘rebound effect’ was observed.

## Conclusion

In this study, we investigated how repeated memorization affects the advantage of memory-matching (relative to mismatching) visual input in gaining conscious access, as memoranda presumably transit from VWM maintenance to LTM storage and back. Our paradigm allowed for VWM usage to vary, while keeping both physical stimulation and task-instructions equal. Here, we questioned whether the prospective reinstatement of VWM usage would revive the accelerated conscious access of memory-matching visual input. Our data showed that the advantage of memory-matching visual input in gaining conscious access is reinstated when observers plan to memorize a novel item imminently. This suggests that, in the anticipation of having to memorize a new item, the VWM machinery is reinstated, thereby incidentally reviving an activity-based VWM representation of the current memorandum. Taken together, our conscious perception is increasingly resilient to mnemonic influences as the same information is repeatedly memorized, but when we anticipate to memorize novel information imminently, our conscious perception suddenly becomes vulnerable to mnemonic influences again.

## Data Availability

Raw data, analysis files and experiment scripts are available here (https://osf.io/n7gpx) via the Open Science Framework.
